# Evaluation of Balance Disorders in Parkinson's Disease Using Simple Diagnostic Tests—Not So Simple to Choose

**DOI:** 10.3389/fneur.2018.00932

**Published:** 2018-10-31

**Authors:** Karolina Krzysztoń, Jakub Stolarski, Jan Kochanowski

**Affiliations:** Department of Neurology of the Second Faculty of Medicine with the English Division and the Physiotherapy Division, Medical University of Warsaw, Warsaw, Poland

**Keywords:** parkinson's disease, balance diagnostics, balance evaluation, balance disorders, balance evaluation systems test, clinical recommandations

## Abstract

**Background:** Balance disorders are one of the main symptoms in parkinson's Disease (PD)—patients have a tendency to fall, related traumas and also a significant restriction of mobility. Numerous tools may be used to evaluate the balance, but it is difficult to choose the proper one. The aim of this review was to compare simple diagnostic tools for PD and emphasize those characterized by a high reliability and sensitivity.

**Methods:** The global literature search was conducted in PubMED, Scopus, Science Direct, Web of Science, Cochrane, and Google Scholar for publications in English and Polish.

**Results:** According to the literature some scales and functional tests in which clinimetric properties had been assessed in PD population were selected and described.

**Conclusion:** Basing on current knowledge, psychometric properties, and clinical experience, the authors suggest the BESTest with its shortened versions and the Fullerton Advanced Balance Scale to be used for comprehensive balance assessment of parkinson's disease patients. These tests are easy in administration, not time-consuming and provide a professional diagnosis allowing to plan individual therapy for the patient being examined.

## Introduction

Postural instability is one of the cardinal signs of Parkinson's disease and an independent risk factor for falls, related traumas and also a significant restriction of mobility (1, 2).

Numerous tools may be used to evaluate balance. Clinical tests and balance scales should be sensitive to various dimensions of postural control including: static and dynamic postural control, sensory orientation, feedforward/feedback postural control reactions (3).

However, there is no standard balance assessment for PD individuals. The pull-test is a part of the Unified Parkinson's Disease Rating Scale (UPDRS) which is most commonly used in clinical practice. However, it is not the best tool, especially if the goal involves the identification of all possible balance problems and the resultant causes (4).

In every day practice clinical tests of balance severity, also in PD, are the Berg Balance Scale (BBS) and Timed Up and Go (5, 6), which are frequently used by clinicians because they require minimal personnel involvement and basic equipment. However, they often have some shortcomings and may not detect relevant impairments of balance control (7, 8).

The present report aimed at describing the value of common diagnostic tests which are used in balance assessment in PD patients. Furthermore, authors chose tools which do not need special, expensive equipment and advanced training to administer. In addition to the tools used in PD some clinimetric properties measured in PD were discussed. Moreover, the authors discussed motor evaluation tools which are characterized by a high sensitivity and may be commonly used by physiotherapists and physicians to evaluate balance disorders in PD. The present paper includes a comparison of various approaches toward balance measurement and their advantages and disadvantages.

The characteristics of various diagnostic tools are presented below in the text and in Tables 1, 2.

**Table 1:** Comparison of selected diagnostic tools in PD.

**Test/Scale name (abbreviation)**	**Number of items**	**Time of administration (min)**	**Interrater/retest reliability**	**Sensitivity/specificity**	**Floor/ceiling effects**
Activities-specific Balance Confidence Scale (ABC)	16	<10–20	Excellent	Moderate to good	No
Balance Evaluation Systems Test (BESTest)	36	20–40	Good to excellent^*^	Good	No
Berg Balance Scale (BBS)	14	15–20	Good to excellent^*^	Moderate	Yes
Fullerton Advanced Balance Scale (FAB)	10	<10	Good to excellent^*^	Good	No
Functional Reach Test (FRT)	1	<10	Poor to excellent^*^	Adequate to good	No
Mini-BESTest	14	10–15	Good to excellent	Adequate to good	No
Timed Up and Go (TUG)	1	<10	Adequate to excellent	Good	Possible
Tinetti Balance Scale	9	<10	Good	Good	Yes

* *differences between study groups e.g., fallers and non-fallers*.

**Table 2:** Comparison of selected diagnostic tools in PD in terms of the components of balance assessment.

**Test/Scale name (abbreviation)**	**Static balance**	**Dynamic balance**	**Sensory orientation**	**Feedforward/feedback**
Activities-specific Balance Confidence Scale (ABC)	–	–	–	–/–
Balance Evaluation Systems Test (BESTest)	+	+	+	+/+
Berg Balance Scale (BBS)	+	+	+	+/–
Fullerton Advanced Balance Scale (FAB)	+	+	+	+/+
Functional Reach Test (FRT)	+	–	–	+/–
Mini-BESTest	+	+	+	+/+
Timed Up and Go (TUG)	+	+	–	+/–
Tinetti Balance Scale	+	+	+	–/+

## Activities-specific balance confidence scale [ABC scale, 1995 (9)]

Activities-Specific Balance Confidence (ABC) Scale is a subjective measurement of balance confidence rated for various ambulatory tasks without falling or feeling unsteady. It is a 16-point subjective questionnaire which rates the patients' balance confidence in daily living situations/activities. The elements are rated on a scale from 0 to 100 with zero points meaning lack of self-confidence and the result of 100 meaning complete confidence. The total number of points is calculated by adding the results of individual items and then dividing by the total number of tasks (9).

Psychometric properties were assessed repeatedly in PD patients (10–13), mostly during the mild to moderate phase of the disease. An excellent test-retest reliability was reported in some studies and moderate to good sensitivity and specificity in others. No floor or ceiling effects were found. However, a poor to adequate correlation between ABC and functional clinical tests was calculated (10). Advantages: the ABC scale is simple to administer, not time-consuming, it does not require any special equipment and relates to daily life activities. Limitations: a subjective evaluation of the patients is always connected with a higher risk of errors; the scale is not strongly correlated with the majority of functional balance test results and does not give information about the type of balance problems. Moreover, the scale is not applicable in cognitive impaired patients.

The ABC Scale is recommended by Neurology section of the American Physical Therapy Association (1–3 H&Y) and The Movement Disorders Society Rating Scales Committee (14).

## Berg balance scale [BBS, 1992 (15)]

The BBS is a 14-item objective measurement tool designed for balance assessment in adult populations while performing common everyday life activities, such as sitting, standing, and changing position. BBS scoring is based on rating the patient's ability to perform each task independently (sometimes with time or distance requirements). The score for each task ranges from 0 to 4, with 4 indicating the highest level of function. Therefore, the patient may score 56 points in total (15).

The BBS was assessed quite frequently in PD patients, mostly in mild to moderate disease (11, 16–18). The scale is relatively safe and simple to implement. However, some equipment is required. Interrater reliability [ICC = 0.84 (17); 0.95 (19)], test-retest reliability (ICC = 0.80) and internal consistency (Cronbach's alpha = 0.92–0.95) were reported to be excellent in some research (17, 20). Floor effects may occur at stages 4 and 5 of H&Y. Moreover, the ceiling effect is present at the early stages of H&Y which was shown in several studies. Therefore, they may prove inadequate for balance assessment at the early stages of postural instability in PD (5, 16, 19, 20). Leddy et al. (19) showed a moderate reliability of the BBS. However, the sensitivity and specificity to discriminate between fallers and non-fallers may not be particularly good. The BBS was designed to measure balance in elderly people, but not specifically in Parkinson's disease patients. Both Leddy et al. (19) and Duncan et al. (21) suggested that the BBS is not a very effective tool for evaluating patients with PD as it does not identify those who are at a risk of falling. However, the Movement Disorders Society Rating Scales Committee recommend the BBS (14). The limitations of BBS are: the presence of the ceiling effect; it is not a comprehensive test—assessing mostly static balance, the missing value addressing reactive postural control—and some equipment is required. The advantages of the Berg's Scale are: easy to administer and not time-consuming.

## Balance evaluation systems test [BESTest, 2008 (22)]

The BESTest is a 36-item clinical balance assessment tool, designed to assess balance disorders. The evaluation process consists of six subscales/sections, each assessing a different aspect of postural control: mechanical constraints, limits of stability, anticipatory postural adjustments, postural response to induced loss of balance, sensory orientation, and gait (22). Each item is scored by the examiner from 0 (the worst) to 3 (the best). The sum of all scores is the total result. However, each category gives its own result, which makes it very helpful in balance assessment to find out which mechanisms of postural control are impaired. The authors provide paper and video instruction, which greatly facilitates correct administration of the BESTest. According to the authors of the test a complete assessment should last about 30 min. However, in clinical practice the evaluation of patients with PD using the BESTest may even exceed 40 min.

There are two shorter versions of the BESTest—the Mini-BESTest and the Brief-BESTest—less time-consuming assessment tools, which may have a higher clinical utility. The Brief version has 1 item from each category of the BESTest (6 items in total).

The assessment of clinimetric properties in PD patients was conducted mostly for the BESTtest and the Mini-BESTest (3, 19, 22–27). The BESTest has an excellent interrater reliability and a good intrarater reliability, face validity and internal consistency. Leddy at al. reported that its comparison with the FGA or BBS, showed that the BESTest has the highest sensitivity to identify between fallers and non-fallers (19). No floor or ceiling effects were reported in the Mini-BESTest. Moreover, it is characterized by a high internal consistency, an excellent interrater reliability and a good intrarater reliability. Moreover, the sensitivity to distinguish between fallers and non-fallers is estimated from adequate to good (3, 21, 25, 27, 28). However, psychometric properties were evaluated mostly in mild to moderate PD cases.

Furthermore, the Brief-BESTest had the highest ability to identify falls in a retrospective cohort study in the elderly (29) and patients who suffered from such diseases as chronic obstructive pulmonary disease (30). However, it is recommended to study all clinimetric properties of the shorter versions of the BESTest on a larger sample of PD patients.

Several advantages of the BESTest have to be emphasized. All the versions of the BESTest are quite simple to administer and are characterized by a high sensitivity. They provide a good evaluation of all balance problems and, importantly, determine the underlying causes of balance disorders. However, the fact that the BESTest requires some equipment and is much more time-consuming than other versions needs to be noted as a limitation.

## Fullerton advanced balance scale [FAB scale, 2006 (31)]

Both static and dynamic balance under varying sensory conditions are tested with the FAB Scale. The scale was designed to measure balance in higher-functioning active elderly adults. The FAB consists of 10 items (each with a 5-point ordinal scale) that require static and dynamic postural control. The range of total score is 0 to 40 points (the higher, the better). Administration instructions may be found in the original paper (31).

The majority of clinimetric properties were assessed in PD patients only once, in mild to moderate disease. The FAB has an excellent test-retest reliability in PD (3), the ceiling effect was not reported (27). There are few studies where the FAB was used to assess postural control in PD, although it was mostly implemented in the same laboratory (3, 26, 27, 32).

The advantages are as follows: the FAB scale is simple to administer, not time-consuming (quicker to perform than BBS and the Mini-BESTest) and allows to assess patients under daily activity conditions to identify the efficiency of all postural control aspects. However, there is contradicting information about the sensitivity and specificity to discriminate between fallers and non-fallers in PD (3) which may be considered a limitation of the scale. The FAB is not recommended by The Movement Disorders Society Rating Scales Committee (14), due to the lack in the clinimetric evidence in PD.

## Functional reach test [FR, 1990 (33)]

The Functional Reach Test (FR or FRT) was designed to assess balance in adult populations by measuring the maximum distance a person can reach forward while standing in a fixed position (33).

Clinimetric properties were assessed in PD patients mostly with the use of 1–3 Hoehn and Yahr score (11, 34–37). According to Lim et al. (34) the interrater reliability was ICC = 0.64. Test-retest reliability was reported as excellent in some research (ICC = 0.84–0.93), mostly for patients with a history of falls and poor (ICC = 0.42) for non-falling subjects (11, 35). However, Kerr et al. (8), reported a significant difference in Functional Reach Test scores between faller and non-faller Parkinson's disease patients (*p* < 0.05). In the report of Behrman et al. (38) the sensitivity of FRT for the risk of falls was only 30%. However, a positive predictive value for persons who actually did have a positive history of falls was 90%, although 44% of the persons at risk remained unidentified by the FRT. In case of patients who had no history of falls and their FRT showed a negative fall risk with the specificity at 92%.

The advantages of the FRT include its simple administration and the fact that it requires basic equipment. However, there is contradictory information about the sensitivity and specificity to discriminate between fallers and non-fallers in PD, which is a limitation of this test. The FRT is recommended by Neurology section of the American Physical Therapy Association (2–3 H&Y) and The Movement Disorders Society Rating Scales Committee (14).

## Push and release test [2006 (39)]

This test was developed as an updated version of the retropulsion test or pull test. According to Foreman et al. the pull test failed to predict falls in PD patients (40). Moreover, the Push and Release test provides a more sensitive and consistent assess of postural stability than the Pull Test in PD population (39, 41). In the Push and Release Test an examiner puts hands on the back of the patient and she or he leans back pressing on the hands of the therapist who suddenly removes them. The result (from 0 to 4) depends on the postural response of the patient (0–the best and 4–the worst response, often with a final fall). The test is characterized by an adequate interrater reliability, an adequate to excellent validity, a high sensitivity, and specificity to discriminate between fallers and non-fallers (39, 41). The advantages of the Push and Release Test include simple administration, time-effectiveness. Furthermore, it requires no special equipment. Limitations: only one item is assessed, so it is not a comprehensive test and does not reflect the type of balance problems. The Push and Release Test is recommended by Neurology section of the APTA and is administered as a part of the UPDRS.

## Tinetti balance scale [TBS, 1986 (42)]

The Tinetti-test was published by Mary Tinetti to assess the gait, balance and risk of falling in the elderly. It is also known as performance-oriented mobility assessment (POMA) or Tinetti Mobility Test (TMT). The scale takes about 10 min to complete. The patient is asked to complete 16 functional tasks 9 of which refer to balance assessment. The patient may score the total of 16 points (42).

According to Contreras et al. the results of Tinetti Balance Scale are correlated with the occurrence of falls (43). Interrater reliability was reported as excellent in some research (ICC = 95%, irrespective of the age and experience of the researcher) (44). Floor effect was found at stages 4 and 5 of Hoehn & Yahr (45). In the assessment of the Korean version of TMT the interrater reliability of the balance evaluation section ranged from 0.94 to 0.98 with an ICC = 0.97 and the respective test-retest reliability, used as a measure of interrater reliability, was ICC = 0.97. Both results are regarded as excellent. The balance part score of 14 was selected as the cut off score with the highest sensitivity (81%) and specificity, which might be useful for predicting falls among PD patients (75%) (46).

The advantages of the scale include simple administration and the necessity to use only basic equipment. The disadvantages include limited information about clinimetric properties in PD, and the presence of the floor effect.

Another version of this scale—Tinetti Falls Efficacy Scale—was used in some research as a scale to measure a tendency to fall. However, it is not commonly used in PD population (46). This version of the TBS is suggested by the Movement Disorders Society Rating Scales Committee, due to the lack of clinimetric evidence in PD (14).

## Timed up and go (TUG, 1991)

The patient is instructed to sit on the chair and place his/her back against the chair. Next, the patient is asked to walk 3 meters at a normal speed, followed by turning around and walking back at a comfortable and safe speed. A stopwatch is used to measure the time between the start and the finish of the test—when the patient's buttocks touch the seat. The test was designed to measure mobility in elderly people and has been advocated as a useful tool for quantifying locomotor performance in people with PD. TUG is characterized by an adequate to excellent test-retest reliability, an excellent interrater reliability, good **s**ensitivity and specificity in predicting the risk of falls (6, 11, 47). Simple administration, time-effectiveness and no need for any special equipment are the advantages. Its main disadvantage results from the fact that it is not a comprehensive test, as it does not reflect the exact type of balance problems—such as evaluating feedforward but not feedback postural control aspects. The TUG is recommended by the Movement Disorders Society Rating Scales Committee (14).

## Discussion

Specifying the most relevant diagnostic tool is highly important as it facilitates the assessment of the patient under different balance conditions. Not many of the discussed tests take e.g., dual-task activities under consideration. Interestingly, performing dual-task activities does not improve after pharmacological treatment, so the balance of the patient might be affected even at early stages of the disease (48).

An ideal balance tool should comprise static and dynamic balance control, postural strategies, proprioceptive information, anticipatory postural adjustments, and balance in functional gait tasks (14, 21, 49–56). There are many tools for assessing balance used in PD population (14, 57–60) however some of them do not take into account all aspects of postural control.

The ABC scale has good clinimertic properties, although it evaluates only balance confidence which is important and allow clinicians to predict future recurrent falls in PD population (12) but it does not measure balance control and does not give any information about type of postural disorders. Moreover, in comparison to functional tests, the ABC Scale results may be overestimated (e.g., fear of falling) or underestimated (14). Marck et al. (57) reported the Push & Release test, Tinetti and Berg Balance Scale (BBS) as tools to evaluate postural instability in PD population in the context of risk of falling (57). The Berg has excellent reliability. However, the scale has limitations such as presence of ceiling effects (3, 5, 16, 19, 29) which may be misleading during assessment of patients with mild deficits. Duncan et al. (21) suggested that the BBS is not the best tool to assess balance control in PD population because it does not detect differences in individuals with freezing of gait (FOG) compared to those without. Additionally it was reported that both the BESTest and the Mini-BEST were able to differentiate between groups. Other study—metaanalysis (59)—showed that BBS scale is also able to detect postural control differences between FOG+ and FOG- groups. However, the Mini-BESTest had lower ceiling effects and a higher sensitivity in comparison to the BBS, moreover it is time-efficient. The FAB Scale is characterized by a high inter-rater and test-retest reliability for the assessment of all aspects of postural control in people with PD (3). Tinetti Balance subscale may be a useful tool for assessing most of balance control aspects and the risk of falls in PD (43), however, due to the lack of high quality psychometric properties assessment in PD other scales may be more appropriate.

## Conclusion

According to current knowledge, psychometric properties, score of the proposed scale, and clinical experience, the authors suggest the BESTest with its shortened versions and the Fullerton Advanced Balance Scale to be used for complex balance assessment of Parkinson's disease patients. These tests evaluate all the mentioned aspects of postural control. Furthermore, they are easy in administration, time-effective and provide professional diagnosis allowing to plan individual therapy for the patient undergoing examination.

Moreover, physiotherapists and physicians should remember about dopaminergic medication effect on postural control, so patient evaluation should be considered both during the “on” and “off” phase (61).

## Author contributions

All the authors participated in the critical review of the report and designed the study. KK and JS were involved in data collection and data analysis. KK wrote the report. All the authors commented on the report and approved the final version.

### Conflict of interest statement

The authors declare that the research was conducted in the absence of any commercial or financial relationships that could be construed as a potential conflict of interest.
